# Roles of ATP and SERCA in the Regulation of Calcium Turnover in Unloaded Skeletal Muscles: Current View and Future Directions

**DOI:** 10.3390/ijms23136937

**Published:** 2022-06-22

**Authors:** Tatiana L. Nemirovskaya, Kristina A. Sharlo

**Affiliations:** Institute of Biomedical Problems RAS, 123007 Moscow, Russia; nemirovskaya@bk.ru

**Keywords:** muscle unloading, SERCA, calcium signaling, modeled microgravity, muscle disuse

## Abstract

A decrease in skeletal muscle contractile activity or its complete cessation (muscle unloading or disuse) leads to muscle fibers’ atrophy and to alterations in muscle performance. These changes negatively affect the quality of life of people who, for one reason or another, are forced to face a limitation of physical activity. One of the key regulatory events leading to the muscle disuse-induced changes is an impairment of calcium homeostasis, which leads to the excessive accumulation of calcium ions in the sarcoplasm. This review aimed to analyze the triggering mechanisms of calcium homeostasis impairment (including those associated with the accumulation of high-energy phosphates) under various types of muscle unloading. Here we proposed a hypothesis about the regulatory mechanisms of SERCA and IP3 receptors activity during muscle unloading, and about the contribution of these mechanisms to the excessive calcium ion myoplasmic accumulation and gene transcription regulation via excitation–transcription coupling.

## 1. Introduction

Skeletal muscle has an extreme plasticity, which allows this tissue to rapidly undergo changes under conditions of increased or decreased mechanical load and activity. A decrease in muscle contractile activity, or its complete cessation, leads to muscle atrophy, fibers’ cross-sectional area (CSA) decrease, as well a reduction in maximum strength, and the speed of muscle contraction and decreased muscle performance [1]. Belova et al. (2020) showed that even a three-week restriction of mobility of rats in the cages of limited size leads to skeletal muscle atrophy, changes in cellular signaling pathways and accelerated weight gain in animals [2]. Now, amidst the COVID-19 pandemic, the look into the causes of these changes is especially relevant, because a large population of people suffer from limited physical activity, which negatively affects their quality of life.

Calcium ions are one of the most important components of cellular signaling in skeletal muscle. Excessive calcium accumulation in the sarcoplasm can lead to fibers’ atrophy and even death, mitochondrial inactivation, and decreased skeletal muscle performance [3]. Various calcium channels in skeletal muscle make it possible to regulate the concentration of calcium in the sarcoplasm, as well as within the myonuclei and mitochondria [4]. The most well-studied calcium channels in skeletal muscles are dihydropyridine, ryanodine, and inositol triphosphate receptors (DHPR, RyR, IP3R). Calcium ions enter the sarcoplasm from the sarcoplasmic reticulum (SR) in response to muscle membrane depolarization via DHPR/RyR coupling mechanism. IP3R (inositol 1,4,5-triphosphate receptors), in addition to Ca^2+^ uptake into the sarcoplasm, also regulate the myonuclear uptake of calcium [4,5]. Changes of calcium concentration in various compartments of muscle fibers remodel the activity of calcium-binding proteins, which in turn alters the functioning of calcium-dependent signaling pathways and gene expression patterns [6]. Removal of calcium ions accumulated in the sarcoplasm as a result of muscle contraction is carried out mainly due to the work of the calcium ATPase of the sarco-endoplasmic reticulum (SERCA) [7].

Various types of muscle unloading lead to the rapid accumulation of calcium ions in sarcoplasm [8,9,10]. Excessive calcium accumulation, in turn, leads to alterations in calcium signaling pathways that contribute to the development of muscle-disuse-induced negative processes in a wide range of muscle disuse types. There are a number of reviews that are mainly focused on the DHPR/RyR function impairment and its consequences under the theme of skeletal muscle aging, disuse, and diseases [11,12]; however, less attention is paid to the roles of SERCA and “slow” calcium signaling under various models of muscle disuse both in reviews and in experimental articles. So-called “slow” calcium signaling has been discovered in recent years. The “slow” Ca^2+^ can stimulate the activation of intracellular signaling pathways and muscle atrophy, and is also involved in excitation–transcription coupling [6,13]. The term “slow” calcium refers to Ca^2+^ ions, the increased concentration of which stays in the sarcoplasm for a long time. The hypothesis about the role of the calcium-dependent regulation of gene expression under skeletal muscle unloading was put forward by S. Kandarian (2002) [14]. Mechanisms of the regulation of genes expression by calcium and IP3R via excitation–transcription coupling in adult skeletal muscle fibers have been described [6,15].

This review will describe several hypotheses about the causes of the unloading-induced calcium ion sarcoplasmic accumulation and pay heed to the regulation of SERCA and a possible role of IP3R under muscle unloading conditions. The review will also analyze the interplay between calcium signaling and macroergic-phosphate-dependent signaling in unloaded skeletal muscles.

## 2. Calcium Homeostasis Alterations in Skeletal Muscles during Unloading

Skeletal muscle unloading is characterized by decreases in muscle contractile activity and/or mechanical load. Skeletal muscle unloading models include rodent hindlimb suspension, muscle immobilization, space flight, human bed rest, and dry immersion [16,17]. Skeletal muscle activity is directly linked to the ATP/ADP ratio; this process is why unloading leads to ATP/ADP ratio increase in muscle. The unloading-induced negative changes are observed in both animals and humans [17]. Calcium ion concentration alterations in unloaded skeletal muscle were shown to play a role in all the key negative consequences of muscle unloading, such as atrophy, force decrease, protein degradation, and even slow-to-fast fiber-type transformation [18,19,20].

Skeletal muscle SR is the main storage as well as the main dynamic regulator of sarcoplasmic calcium concentration in the muscle [21]. The interaction between the SR and the T-tubules is critical for electro-mechanical or excitation–contraction coupling (ECC) [22]. In skeletal and cardiac muscles, different ECC-mediating calcium channels are expressed: in skeletal muscles, they are Ca V 1.1 and RyR1, while in cardiac muscles they are Ca V 1.2 and RyR2. SR is the source of calcium ions for the activation of the muscle sarcomeres [7]. In skeletal muscle, unlike the heart, DHPR activates RyR by direct mechanical connections in the muscle triad (the triad is a structure formed by the T-tubule region and the nearby two SR terminal cisternae). Muscle RyR are located in the terminal cisternae SR membrane, while DHPR are located in T-tubule membrane [22]. The activation of RyR leads to the release of calcium from SR, the formation of myosin–actin cross-bridges, and muscle contraction. Reuptake of this calcium to the SR is the necessary step to stop muscle contraction. Removal of calcium ions from the sarcoplasm is carried out by four main types of calcium transporters: the Na^+^/Ca^2+^ exchanger, sarcolemmal Ca^2+^-ATPase, Ca^2+^ mitochondrial uniporter, and Ca^2+^ sarcoplasmic reticulum ATPase (SERCA) [23,24]. Among them, SERCA plays a key role in removing calcium ions from the sarcoplasmic reticulum and provides the calcium storage of the SR SERCA makes up approximately 80% of the total protein fraction of the longitudinal SR [7,25,26,27] and consumes 40–50% of ATP in resting skeletal muscle [28].

During muscle unloading, the regulation of calcium homeostasis in muscle fibers undergoes rapid and dramatic alterations. For example, in a rodent hindlimb unloading model, the increase of the resting sarcoplasmic calcium ion concentration in soleus muscle occurred as early as after the second day of the experiment and remained elevated up to the 14th day [8,9]. There is indirect evidence of calcium ion accumulation even after 12 h of rat hindlimb suspension in the mixed vastus muscle [29]. The increased calcium ion content was also observed after 4 weeks of immobilization of the soleus and gastrocnemius muscles in rats and the soleus and plantaris muscle in mice [10,30]. The detailed causes of the unloading-induced calcium ion accumulation are unclear, however, a number of studies show that the changes in the activities of DHPR, RyR, and probably the inactivation of SERCA are all involved in the unloading-induced impairment of calcium homeostasis [8,9,26,31,32,33]. So, how do these channels contribute to the unloading-induced calcium ion sarcoplasmic accumulation and what is the role of macroergic-phosphate-dependent signaling in this process?

The observation of an early-stage muscle membrane depolarization provides a base for a hypothesis about the role of DHPR in the unloading-induced calcium ion accumulation. In 2015 it was demonstrated that 24–72 h of rat hindlimb suspension specifically decreases the electrogenic activity of the Na,K-ATPase α2 isozyme and the resting membrane potential of soleus muscle fibers [34]. In 2021 the same research group detected the decrease in resting membrane potential of soleus muscle fibers as early as after 6 h of rat hindlimb suspension, and preventing Na,K-ATPase dysfunction rescues the resting membrane potential of soleus muscle fibers [35]. Earlier it was shown that the unloading-induced decrease in the resting membrane potential of soleus muscle fibers is accompanied by the sarcoplasmic accumulation of calcium ions after 3 days of unloading [32]. Pharmacological blocking of DHPR channels by nifedipine prevented this accumulation [32]. The blocking of DHPR during 3 days and 14 days of rat hindlimb suspension also prevented the accumulation of calcium ions in the soleus sarcoplasm, led to the partial prevention of slow-to-fast muscle fiber-type shift, as well as the upregulation of proteolysis [18,19,20]. As DHPR are the voltage-sensitive channels, it can be suggested that inactivation of Na,K-ATPase at an early stage of rat hindlimb suspension and the subsequent decrease in muscle resting membrane potential are a triggering mechanism for DHPR opening, leading to the sarcoplasmic accumulation of calcium ions [32].

However, it is still unclear what are the causes of the unloading-induced Na,K-ATPase disfunction.

It was found that Na,K-ATPase in skeletal muscle can be activated by AMP-activated protein kinase (AMPK) [36]. AMPK activity is decreased dramatically during the early stage of rat hindlimb unloading (1–3 days) [37,38] and activation of AMPK by AICAR has a protective action on the soleus muscle fibers’ resting membrane potential after 12 h of rat hindlimb suspension [39]. Low-dose ouabain prevents both Na,K-ATPase and AMPK inactivation in soleus muscle during 6 h of rat hindlimb suspension. So, there is a possible functional link between AMPK and Na,K-ATPase inactivation [35]. The hindlimb suspension-induced AMPK inactivation is in turn triggered by an increase in the ATP/ADP ratio, which occurs as the result of soleus muscle contractile activity cessation at the early stage of the hindlimb suspension model of muscle unloading [1,40,41]. So, summing it all up, it can be concluded that the decline in muscle electrical activity leads to the accumulation of high-energy phosphates, which inactivates AMPK. AMPK inactivation leads to Na,K-ATPase inactivation, a decrease in muscle membrane resting potential, the opening of voltage-dependent DHPR, and sarcoplasmic calcium ion accumulation. This mechanism was described in the model of rodent hindlimb suspension; however, it can contribute to other unloading models.

Another set of experimental data show that the SR-sarcoplasmic traffic of calcium ions is also dramatically impaired under various models of muscle unloading. For example, in the rat denervation model, the transport of calcium ions from the sarcoplasm to the SR in the slow skeletal fibers of the soleus muscle is significantly reduced at the early stages of the experiment (2 days) [33]. During immobilization of the lower limbs in humans, there was also a decrease in calcium ions transport from the sarcoplasmic to the SR of the vastus lateralis muscle on days 3, 6, and 10 of the experiment [31]. The accumulation of calcium ions and the changes in their reuptake from the sarcoplasm into the SR was found in the muscles of humans and animals for different models of unloading (hindlimb suspension, denervation, immobilization) [8,33,42,43].

Sarcoplasmic calcium accumulation during unloading leads to various signaling changes, involving feedback inhibition. For example, calcium–calmodulin kinase (CaMKII) is regulated by the intracellular calcium concentration [44,45]. Calcium accumulation, leading to CaMKII activation, was previously reported upon muscle unloading [8,9]. CaMKII regulates the phosphorylation of numerous proteins, including AMPK and a number of transcription factors [46]. CaMK II also phosphorylates calcium-dependent phosphatase calcineurin, decreasing its activity [47].

Interestingly, in an in vivo experiment, both calcium uptake and calcium release (by passive leakage) from the SR were enhanced in the soleus muscle upon hindlimb-suspended rats, while in the extensor digitorum longus muscle there was enhanced calcium passive leakage only [48].

The causes of these alterations can be explained by the hypothesis of “leaky” RyR channels and by alterations in SERCA functioning. DHPR, during prolonged membrane depolarization, can regulate the calcium traffic capacity of RyR in the unloaded skeletal muscles. This mechanism of calcium “leakage” from the SR due to the activation of ryanodine channels was found in the type I muscle fibers of elderly people, as well as in beta-sarcoglycan knockout mice, in mdx mice, and even in the diaphragm during mechanical ventilation [49,50]. Andersson et al. hypothesized a presence of a self-sustaining cycle of calcium leakage from the SR: the authors suggest that excessive calcium release can lead to nitrosylation and oxidation of the ryanodine receptor subunits and detachment of calstabin-1 from the ryanodine receptor; calstabin-1 is a protein required to maintain the ryanodine receptor in closed state [49]. Destabilization of the ryanodine receptor, in turn, leads to calcium ion accumulation in the sarcoplasm [50]. It can be assumed that the self-sustaining mechanism of calcium leakage from the SR described by Andersson et al. may also take place during unloading, contributing to the excessive accumulation of calcium ions in the sarcoplasm. However, it is still unclear what prevents the return flow of calcium ions from the sarcoplasm into the SR if there is a mechanism for its return transport by SERCA.

## 3. Changes in SERCA Expression and Posttranslational Regulation during the Unloading of Muscles

SERCA is a P-type transmembrane protein [7]. In mammals, three SERCA genes are expressed: SERCA1, SERCA2, and, to a minor extent, SERCA3. More than ten mRNA isoforms are synthesized on the template of these genes as a result of alternative splicing. Isoforms 1a, 2a, and 2b are expressed in the skeletal muscle of an adult mammal. The structure and regulation of SERCA function have been described [7,51]. At the same time, the number of studies investigating the work of SERCA in the heart muscle is much higher than in the skeletal muscle, and there are few studies investigating SERCA during muscle unloading. What changes does SERCA undergo under muscle unloading?

Fibers of the “slow” type are characterized by a predominance of the “slow” isoform of myosin heavy chains, as well as the isoform of SERCA2a, while the “fast” fibers contain fast isoforms of myosin heavy chains and the SERCA1 isoform (SERCA1b is expressed in neonatal muscles and SERCA1a in adult muscles) [52]. SERCA 2b is expressed in both fast and slow fibers [53]. It was previously shown that in humans and rodents, in the various models of unloading of skeletal muscles, after the first week of unloading, and sometimes even earlier, slow-to-fast fiber type transformation was observed [54,55,56,57,58]. A change in the relative SERCA1 and SERCA2a-positive muscle fiber percentage precedes a change in the percentage of slow and fast myosin heavy chain-positive muscle fibers (accessed by immunohistochemistry) [42]. Both muscle hindlimb suspension and denervation led to a decrease in SERCA2a expression and an increase in SERCA1 expression [59]. In one study it was also shown that there was an increase in SERCA2b expression under 7 days of female rat hindlimb suspension in the soleus muscle [60]. Studies of the activity of SERCA isoforms in vitro showed that the rate of SERCA1 activity is twice that of SERCA2a when normalized to the isoforms’ level of expression [61]. Based on this fact, it could be assumed that SERCA should be more active in the unloaded muscle fibers because of the unloading-induced slow-to-fast SERCA isoform expression transformation and increased SERCA2b expression. However, experimental data do not support this suggestion [30,62]. So, the changes in SERCA isoforms expression pattern are not sufficient to predict the level of SERCA activity under muscle unloading conditions. At the same time, it should be kept in mind that SERCA activity can be regulated not only by expression but also by post-translational and allosteric modifications.

The allosteric regulation of SERCA is carried out through interaction with endogenous inhibitors and activators. Of these, the proteins sarcolipin and phospholamban are the most well studied. In addition, there is evidence of activating post-translational modifications of SERCA, such as acetylation, sumoylation, and S-glutathionylation [63,64,65,66].

When bound to phospholamban (PLN) and sarcolipin (SLN), SERCA reduces its activity [51,67,68], but they inhibit SERCA only when unphosphorylated [69]. The mechanism of the interaction of these proteins with SERCA has been described in detail [7,70]. PLN and SLN are transmembrane proteins, and both are able to bind to SERCA isoforms 1 and 2, form heterodimeric complexes, and reduce the affinity of SERCA for calcium [68]. According to some reports, in addition to this effect, SLN is also able to reduce the maximum velocity of the enzyme [68]. SLN, unlike PLN, is able to uncouple ATP hydrolysis and SERCA calcium transport, which leads to heat generation in muscles [71]. The regulation of phospholamban activity is better studied in cardiac muscle: it has been shown that the phosphorylation of phospholamban at the 16 serine residue and 17 threonine residue by protein kinase A and calcium-dependent protein kinase II, respectively, leads to inactivation of phospholamban and the activation of SERCA [72,73]. There is evidence that phospholamban and sarcolipin influence each other: the phosphorylation of sarcolipin facilitates the subsequent phosphorylation of phospholamban and the activation of SERCA [51]. At the same time, it was hypothesized that PLN plays a different role from SLN in skeletal muscle [74].

Some time ago it was declared that PLN is expressed only in “slow” skeletal fibers, and SLN only in “fast” ones [75,76]. If so, the expression of PLN and SLN should be changed under muscle unloading due to slow-to-fast fiber-type transformation. However, PLN expression in slow skeletal fibers varies widely from one mammalian species to another [77,78]. Moreover, in rodents, phospholamban is also found in “fast” muscle fibers [79]. At the same time, it was found that sarcolipin and phospholamban are co-precipitated in the fibers of the vastus lateralis muscle in humans, so it can be concluded that both proteins are expressed in the same fiber [80].

There is not much information about the details of posttranslational SERCA regulation by SLN and PLN under muscle unloading. Upon 37 days of exposure to microgravity, it was observed that there was a decrease in PLN and an increase in the SLN contents in the soleus muscles of mice. These changes were accompanied by alterations in calcium ion SR uptake and increased levels of total protein T-nitration and S-nitrosylation. At the same time, in the tibialis anterior muscles of the experimental animals, the levels of total protein T-nitration and S-nitrosylation as well as calcium ion SR uptake were unaltered [62]. The authors of the described research assumed that SERCA function during space flight may be impaired due to its T-nitration and/or S-nitrosylation [62]. As it was indicated that S-nitrosylation of PLN and SLN can also affect the work of SERCA, this mechanism can also contribute to the observed changes [81].

In the experiment using 2-week muscle denervation, there was a simultaneous increase in PLN expression and a decrease in SERCA activity in mice in the tibialis anterior and flexor digitorum brevis [82]. The authors of that study noted that an abnormal increase in PLN and a decrease in SERCA activity were accompanied by a change in the level of calcium ions in the SR, as well as a change in the peak calcium content and the muscle contraction strength of denervated muscles [82]. It is interesting to note that in the work of Tomiya and co-authors [30], upon immobilization of mouse limbs, the expression of PLN and SLN in the soleus and plantaris muscle also increased; calcium ion accumulation in the sarcoplasm was recorded, but no change in the expression of SERCA isoforms was observed. Basing on these findings the authors concluded that, under these conditions, the activity of SERCA is regulated not by its isoform composition, but by the post-translational regulation. Evidently, the contribution of SLN and PLN to the unloading induced changes needs further investigations.

Little is known about the causes of SERCA function alteration in muscle unloading.

**Figure 1:**
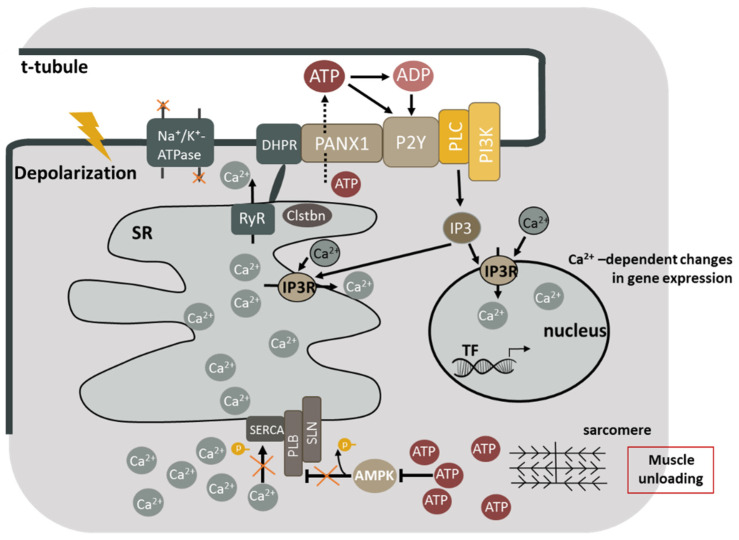
Molecular mechanisms contributing to the sarcoplasmic calcium accumulation during skeletal muscle unloading. Inactivation of Na,K-ATPase leads to sarcolemma depolarization, which in turn causes the activation of DHPR and the opening of RyR. Sarcoplasmic calcium accumulation leads to the detachment of calstabin from the ryanodine receptor and the occurrence of calcium leakage. Meanwhile, the inactivation of AMP-dependent protein kinase leads to dephosphorylation of PLN, and the blockage of SERCA. Excessive accumulation of calcium ions in the sarcoplasm causes calcium-dependent proteolysis and changes in gene expression in muscle fibers. ATP is released by PANX1 under functional muscle unloading. ATP is rapidly degraded to ADP, AMP, and adenosine under the action of ectonucleotidases. ATP and ADP can affect P2Y receptors associated with G proteins that in turn activate PI3 kinase. PI3 kinase catalyzes phosphorylation of phosphatidylinositol diphosphate (PIP2), giving PIP3 a highly charged residue, which recruits phospholipase C into the membrane, triggering the formation of inositol-1,4,5-triphosphate (IP3). IP3 then binds to IP3 receptors (IP3R) present both in the nuclear envelope and in the sarcoplasmic network, causing a weak signal of calcium release both in the cytosol and in the nucleoplasm, which contributes (probably, with other signaling cascades) to the activation of transcription factors (TF) leading to the expression or repression of the genes involved in the phenotype of muscle cells. DHPR—dihydropyridine channels; RyR—ryanodine receptors; SERCA—sarcoplasmic calcium-dependent ATPase; Clstbn—calstabin; AMPK—AMP-dependent protein kinase; PANX1—pannexin channels; P2Y—P2Y receptors; G—g-protein; PI3K—PI3-kinase; IP3R—IP3 receptors (IP3R—inositol 1,4,5-triphosphate receptors); IP3—inositol 1,4,5-triphosphate; PLC—phospholipase C.

However, one mechanism of SERCA inactivation during unloading may be mediated by ATP/ADP-ratio-dependent pathways. It should be noted that the regulation of PLN activity itself can also be carried post-translationally. PLN and SLP inhibit SERCA only when unphosphorylated [7,70]. For example, the phosphorylation of PLN and SLN, which can be carried out by both CaMK II and AMPK, is capable of restoring the activity of SERCA [83,84,85]. AMPK activation by metformin, as well as by A769662, suppresses endoplasmic reticulum stress by increasing the phosphorylation of PLN [85,86]. At the same time, AMPK phosphorylation decreases dramatically after 1–3 days of unloading [1,37,38]. So, the unloading-induced decrease in AMPK phosphorylation may be the reason for a decrease in PLN phosphorylation, which blocks SERCA activity under early stages of hindlimb suspension in the soleus muscle (and in other variants of muscle unloading that are accompanied by muscle activity decrease and ATP/ADP ratio increase). This mechanism can link the accumulation of high-energy phosphates at an early stage of skeletal muscle unloading with the disruption of SERCA functions. A diagram of this mechanism is shown in Figure 1.

In recent years, several micropeptide regulators of SERCA activity have been discovered [87,88]. The first one of them was the sarcolamban peptide found in Drosophila cells and highly conserved in animals from insects to humans [87]. The amino acid sequence of this peptide is encoded in a long non-coding mRNA and was identified by searching for possible short open reading frames (ORFs). Two isoforms of this peptide, consisting of 28 and 29 amino acid residues, are transmembrane proteins and are able to regulate calcium homeostasis in the heart muscle of Drosophila [87]. In 2015, Anderson and co-authors discovered the myoregulin peptide, which consists of 46 amino acid residues. This peptide is expressed in both fast and slow fibers of mouse skeletal muscles, regulated by transcription factors of the MEF-2 and MyoD families, and interacts with SERCA, inhibiting its activity. Myoregulin gene knockout mice an increased muscle performance and an increased traffic of calcium ions in the SR [89].

Recently, a SERCA activator protein called DWORF was discovered, the amino acid sequence of which is also encoded in the open reading frame of a long noncoding RNA [88]. DWORF is expressed in the heart as well as in “slow” type skeletal muscle fibers, including the soleus muscle and diaphragm. This protein binds to SERCA and blocks its interaction with phospholamban. In mice overexpressing DWORF in cardiomyocytes, the accumulation of calcium in the SR, as well as the activity of SERCA, is enhanced [90]. In the absence of the DWORF gene, SERCA is inactivated in “slow” skeletal fibers [88]. To date, DWORF is the only known endogenous SERCA activator.

Apart from endogenous SERCA activity modulators, there are pharmacological methods of SERCA activation. There are several studies using SERCA activators under various conditions, characterized by an excessive accumulation of calcium ions in the sarcoplasm. The use of the SERCA allosteric activator (CDN1163) for 7 weeks in mdx mice led to an increase in muscle strength and performance, as well as to a decrease in muscle degeneration rate [91]. The use of the same pharmacological agent led to a decrease of soleus and gastrocnemius muscles’ atrophy and an increase in muscle performance in superoxide dismutase-deficient mice [92], as well as to decreases in muscle atrophy, mitochondrial dysfunction, and deterioration of muscle performance during the aging of mice [93]. The use of direct or indirect (due to the impact on the endogenous regulators of SERCA) methods of SERCA activation under muscle unloading can be a promising approach to preventing the negative consequences of the excessive accumulation of calcium ions in the sarcoplasm.

## 4. Interconnection of ATP and Calcium-Dependent Processes in Skeletal Muscle during Unloading

There is an interconnection between the ratio of high-energy phosphate levels and calcium-dependent signaling in skeletal muscle during unloading. In unloaded muscles, the ratio of high-energy phosphates changes [94,95]. At the early stage of unloading (3 days), the level of high-energy phosphates in the muscle (in the model of rat hindlimb unloading) increases [96]. During this period, an almost complete absence of electrical and mechanical activity of soleus muscle is usually recorded [40]. Expectedly, under an absence of contractile activity and unarrested mitochondria functioning, ATP content increases. This increase leads to the inactivation of AMP-dependent signaling pathways, including AMP-dependent protein kinase (AMPK), and AMPK inactivation occurs as early as after 12 h of unloading and lasts until the third day [38,94,97,98]. ATP accumulation at the early stages of muscle unloading can influence the sarcoplasmic calcium content by two ways: (a) reducing the level of phosphorylation of AMPK; (b) activating purinergic receptors as a result of the release of ATP through the pannexin channels [6,99,100]; (c) through a series of intermediate cascades associated with P2Y2 receptors, changing IP3 (inositol 1,4,5-triphosphate) receptor activity [101,102]. In an experiment with a human cell culture, it was shown that the import of external ATP and AMP regulates AMPK-dependent signaling pathways and mTORC1 activity [103].

It is known that AMPK associates with the regulation of calcium metabolism and is regulated in a Ca^2+^/calmodulin-dependent manner [44]. Earlier, an interconnection among the accumulation of ATP, nucleoplasmic calcium content, and changes in the activity of calcium-dependent transcription factors in the myonuclei, leading to genes expression changes, was found [101]. It is known that calcium accumulation (as well as high-energy phosphates) in the sarcoplasm during muscle unloading leads to the activation of IP3 receptors [99,104].

Recently, it was found that PLN, which inhibits SERCA channels, can also interact with IP3R, changing its calcium pass-through function and regulating calcium nucleoplasmic content [101,105].

In the laboratory of E. Jaimovich it was shown that exogenous ADP, UTP, and UDP activate transient calcium traffic in the cell. The electrical stimulation of the myotubes showed a rapid increase in extracellular ATP, ADP, and AMP, as well as the induction of free intracellular calcium concentration increase, with an EC50 value of 7.8 ± 3.1 μm by exogenous ATP [106]. IP3R are found in the membrane of the sarcoplasmic reticulum and in the membrane of the nuclei (which also contain calcium storage). Previously, it was hypothesized that a high local concentration of calcium at the nuclear membrane is required for the activation and translocation of calcium-dependent transcription factors (NFAT, HDAC, etc.), including their phosphorylation/dephosphorylation [107]. The mechanism of this process can be as follows: depolarization of the sarcolemma during muscle unloading leads to the opening of dihydropyridine (DHPR, L-type Ca-dependent) channels, which are tightly connected to pannexin channels (Panx1) [101,108]. Muscular ATP enters the extracellular space through pannexin channels (PANX1) (Figure 1) [109,110,111]. These nucleotides can then interact with the purinergic P2Y receptors [106], which in turn activate PI3-gamma kinase (PI3K) (in the triad membranes) and, ultimately, IP3 receptors located in the nucleus and sarcoplasmic reticulum membranes [101]. These authors have previously described the interactions of DHPR, PANX1, purinergic receptors P2Y, G proteins, PLC, and PI3K in the sarcolemma [101]. Previous studies have shown that agonist-activated purinergic receptors promote the release of calcium ions through IP3-dependent and RyR-independent mechanisms [112]. IP3R activity is regulated by Ca^2+^ and IP3 [99,113]. The activation of IP3 receptors (IP3R) can induce a weak signal of calcium release, both cytosolic and nucleoplasmic, which promotes the activation of transcription factors, which in turn leads to the activation or repression of muscle-phenotype-regulating gene transcription [6,15]. The laboratory of Dr. E. Jaimovich has investigated the role of PI3K in this signaling pathway in a series of experiments with skeletal muscle fiber cultures [102,114,115]. Recently, in an experiment with the inhibition of pannexin channels during 1–3 days of rat hindlimb suspension, they found an increase in the level of ATP and a decrease in the expression of E3 ligases (MuRF1 and MAFbx) in the soleus muscle [96]. These results indicate that during skeletal muscle unloading, the ATP-permeable PANX1 channels are involved in the regulation of muscle atrophy, the modulation of E3 ligases expression, as well as the signaling pathways that control the processes of protein translation and elongation. A possible mechanism of operation of the described signaling pathways during unloading is shown in Figure 1.

In the past decade, a number of evidence has been collected suggesting that alterations in Ca^2+^ transport from the endoplasmic reticulum (ER) are mediated by the macromolecular complex formed by IP3R [116]. The authors of the cited study describe the involvement of this mechanism in diseases and skeletal muscle dysfunction (neurodegenerative diseases, Duchenne muscular dystrophy, age-related muscle atrophy, and exercise-induced muscle damage). Although a role of IP3 in the regulation of gene expression can be recognized in muscles, at the present time it is not directly supported by experimental data on muscle hindlimb unloading. In this section, we have shown that IP3 receptors are involved in the regulation of skeletal muscle gene expression in various diseases and skeletal muscle dysfunction, as well as in the cell culture. Since the similar changes in the regulation of calcium metabolism and energy metabolism are also observed upon muscle unloading, we believe that a similar mechanism of transcription regulation of genes with the help of excitation–transcription coupling (as described in Dr. Jaimovich studies) may take place. That is why we would like to draw the attention to this problem as one of the directions for future investigations.

## 5. Conclusions and Future Directions

The excessive accumulation of sarcoplasmic calcium is observed under different states of striated muscle inactivation and leads to a wide range of negative consequences, including the activation of calcium-dependent proteolysis, slow-to-fast fiber-type shift, and functional disorders. Nowadays, the data available in the literature suggest that the main reason for the sarcoplasmic calcium accumulation during muscle unloading is a deregulation in the function of DHPR, RyR, and SERCA. This deregulation leads to an increase in the traffic of calcium ions into the sarcoplasm and a decrease in its reuptake into the SR. The triggering mechanisms of this deregulation can be both depolarization of the sarcolemma in the first hours of muscles unloading, and the accumulation of high-energy phosphates, which inactivates SERCA and nuclear IP3 receptors. It is likely that the activity of SERCA and IP3R receptors can be regulated by AMPK via phosphorylation of PLN and SLP and, thereby, control the concentration of calcium ions in the sarcoplasm. ATP-permeable PANX1 channels are involved in the regulation of muscle atrophy by modulating transcription processes. Further study of the role of endogenous SERCA regulators, as well as the use of direct and indirect methods of SERCA activation under conditions of muscles unloading, is necessary for the development of methods to compensate for the negative consequences of unloading-induced impaired calcium homeostasis.

## Abbreviations

AMPK	AMP-dependent protein kinase
PANX1	pannexin channels
Clstbn	calstabin
CSA	cross-sectional area
DHPR	dihydropyridine channels
ECC	excitation-contraction coupling
EMG	electromyography
G	g-protein
IP3	inositol 1,4,5-triphosphate
IP3R	IP3 receptors (IP3R-inositol 1,4,5-triphosphate receptors)
P2Y	P2Y receptors
PI3K	PI3-kinase
PLC	phospholipase C
PLN	phospholamban
RyR	ryanodine receptors
SERCA	sarcoplasmic calcium-dependent ATPase
SLN	sarcolipin
SR	sarcoplasmic reticulum
